# Effective dominance of resistance of *Spodoptera frugiperda* to Bt maize and cotton varieties: implications for resistance management

**DOI:** 10.1038/srep34864

**Published:** 2016-10-10

**Authors:** Renato J. Horikoshi, Daniel Bernardi, Oderlei Bernardi, José B. Malaquias, Daniela M. Okuma, Leonardo L. Miraldo, Fernando S. de A. e Amaral, Celso Omoto

**Affiliations:** 1 Department of Entomology and Acarology, Luiz de Queiroz College of Agriculture (ESALQ), University of São Paulo (USP), Av. Pádua Dias 11, Piracicaba 13418-900, São Paulo, Brazil

## Abstract

The resistance of fall armyworm (FAW), *Spodoptera frugiperda*, has been characterized to some Cry and Vip3A proteins of *Bacillus thuringiensis* (Bt) expressed in transgenic maize in Brazil. Here we evaluated the effective dominance of resistance based on the survival of neonates from selected Bt-resistant, heterozygous, and susceptible (Sus) strains of FAW on different Bt maize and cotton varieties. High survival of strains resistant to the Cry1F (HX-R), Cry1A.105/Cry2Ab (VT-R) and Cry1A.105/Cry2Ab/Cry1F (PW-R) proteins was detected on Herculex, YieldGard VT PRO and PowerCore maize. Our Vip3A-resistant strain (Vip-R) exhibited high survival on Herculex, Agrisure Viptera and Agrisure Viptera 3 maize. However, the heterozygous from HX-R × Sus, VT-R × Sus, PW-R × Sus and Vip-R × Sus had complete mortality on YieldGard VT PRO, PowerCore, Agrisure Viptera, and Agrisure Viptera 3, whereas the HX-R × Sus and Vip-R × Sus strains survived on Herculex maize. On Bt cotton, the HX-R, VT-R and PW-R strains exhibited high survival on Bollgard II. All resistant strains survived on WideStrike, but only PW-R and Vip-R × Sus survived on TwinLink. Our study provides useful data to aid in the understanding of the effectiveness of the refuge strategy for Insect Resistance Management of Bt plants.

Fall armyworm (FAW), *Spodoptera frugiperda* (J. E. Smith), is the primary pest of maize (*Zea ma*ys L.) and cotton (*Gossypium hirsutum* L.) in South American countries^1^,^2^. In Brazil, some biological characteristics of FAW—such as polyphagy^3^, a high reproductive capacity and adult dispersion^4^, and multiple generations per year^5^,^6^— favor high infestation rates of this pest on maize and cotton throughout the year.

The use of transgenic maize and cotton varieties expressing insecticidal proteins from *Bacillus thuringiensis* Berliner (Bt) is the main control strategy for FAW in Brazil^7^,^8^,^9^,^10^. During the 2014–2015 crop season, Bt maize and cotton were cultivated on approximately 80 and 40%, respectively, of the total area used for these crops^11^. Among the several available commercial Bt maize and cotton varieties, most express Bt proteins from the Cry1 group, with some proteins showing cross-resistance, such as Cry1F, Cry1A.105, Cry1Ac, and Cry1Ab^12^,^13^,^14^,^15^,^16^. In the current Brazilian crop production system, overlap of the cultivation of some Bt plants (i.e., maize, cotton and soybean) occurs, and cross-crop resistance among Bt plants can contribute to the evolution of resistance in field populations of FAW^16^,^17^.

The evolution of resistance in populations of *S. frugiperda* is the primary threat to the sustainability of Bt maize and cotton varieties. In Brazil, FAW has evolved field-relevant resistance to the Cry1F protein expressed in Herculex maize^6^ and to the Cry1Ab protein in MON810 maize^18^. This species has also developed resistance to Cry1F maize in Puerto Rico^19^ and in some areas of the southeastern United States^13^. Laboratory studies have also indicated the resistance of FAW to YieldGard VT PRO maize, which expresses Cry1A.105/Cry2Ab2^12^,^16^; PowerCore maize, which expresses Cry1A.105/Cry2Ab2/Cry1F^20^; and Agrisure Viptera and Agrisure Viptera 3 maize, which express Vip3Aa20 and Vip3Aa20/Cry1Ab^21^, respectively. The laboratory selection of these resistant strains from field populations of FAW is indicative of the presence of resistance alleles in the field, which reveals a potential risk of the evolution of resistance if resistance management strategies are neglected.

The resistance management strategies called ‘high-dose/refuge’ and ‘gene pyramiding’ have been proposed to prevent or delay the evolution of resistance in FAW to Bt plants in Brazil. The first strategy combines refuge areas, where susceptible insects are generated for mating with the rare resistant insects that survive in the Bt areas, with the high-dose expression of Bt protein(s), which causes the resistance to be “functionally” recessive^22^,^23^,^24^. The second strategy uses plants that express two or more Bt proteins with high insecticidal activity against the same target pest^25^,^26^. Theoretically, only the individuals who are homozygous for resistance to all proteins survive on Bt plants under this strategy^27^. Mathematical models suggest that the use of the gene pyramiding strategy more efficiently delays the evolution of resistance compared with the use of plants that express only one insecticidal protein^28^,^29^. In this context, to support resistance management programs, we evaluated the functional dominance of FAW resistance to Bt maize, based on the survival of resistant, heterozygous, and susceptible strains on the Bt maize and cotton varieties used commercially in Brazil.

## Results

### Survival of FAW strains on Bt maize varieties

A significant interaction was present between Bt maize varieties and FAW strains (df = 40, deviance = 1905.00, *P *< 0.0001). The effect of Bt maize (df = 5, deviance = 3536.80, *P *< 0.0001) and FAW strains (df = 8, deviance = 1103.70, *P *< 0.0001) was also significant.

**Figure 1:**
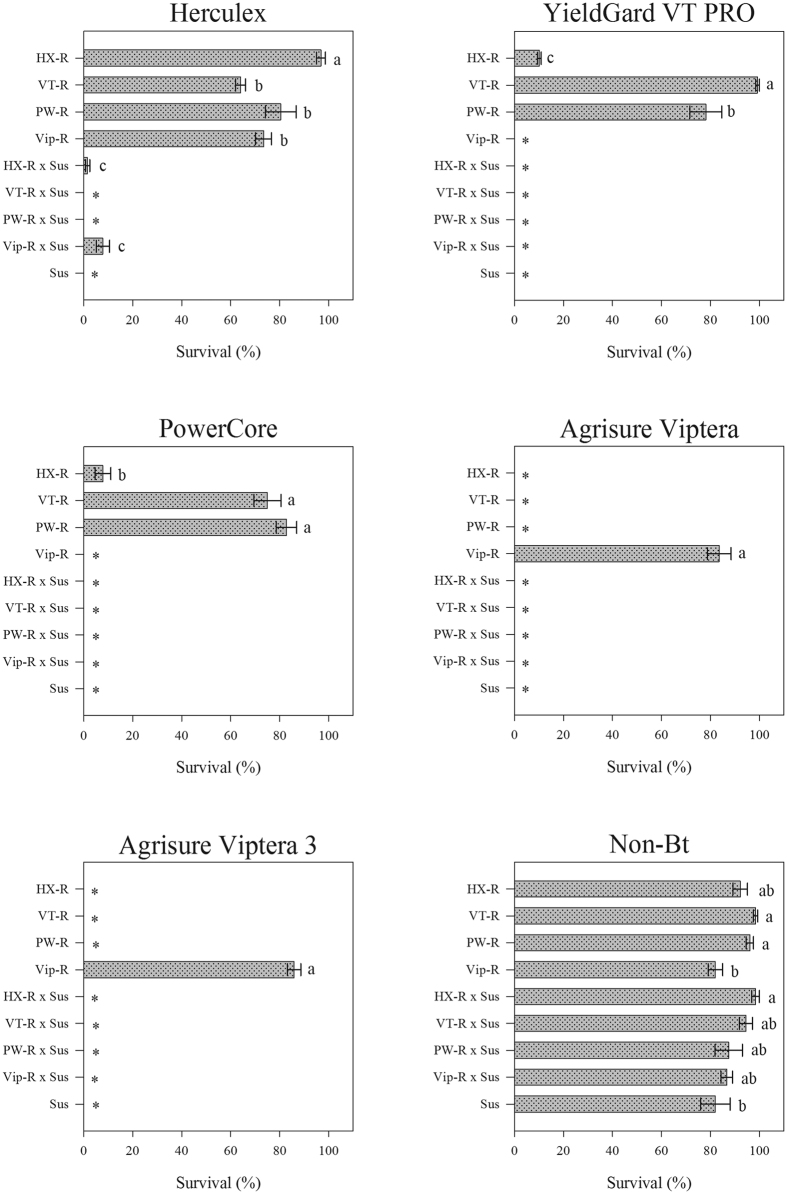
Survival of Bt-resistant, heterozygous, and susceptible strains of FAW on the leaves of Bt and non-Bt maize at 7 days. Bars (±SE) with the same letter are not significantly different when confidence intervals overlap (95% CI). An asterisk (*) indicates that confidence intervals were not estimated because no variability existed.

Figure 1 shows the effect of the Bt maize variety on the survival of the FAW strains. The HX-R strain had the highest larval survival rate on Herculex — greater than 96%. In this Bt maize variety, resistant larvae from the VT-R, PW-R and Vip-R strains had significantly lower survival than did the HX-R strain but were not different from each other. Heterozygous larvae from HX-R × Sus (~2% survival) and Vip-R × Sus (~8% survival) exhibited significantly lower survival than the HX-R, VT-R, PW-R and Vip-R strains on Herculex. In contrast, the Sus, VT-R × Sus and PW-R × Sus strains did not survive on this maize variety (Fig. 1). The VT-R and PW-R strains exhibited significantly higher survival than other strains on YieldGard VT PRO and PowerCore — greater than 75% survival—whereas the HX-R strain had a survival rate less than 11%. In contrast, Sus, Vip-R, and all of the heterozygous strains did not survive on YieldGard VT PRO or PowerCore (Fig. 1). Only resistant larvae from the Vip-R strain survived on Agrisure Viptera and Agrisure Viptera 3 at a rate greater than 83% (Fig. 1). On non-Bt maize, all of the FAW strains had survival rates greater than 82% (Fig. 1).

The larval survival results of the Bt-resistant, heterozygous, and susceptible FAW strains on the leaves of Bt and non-Bt maize after 7 days are presented in Supplementary Table S1. The HX-R strain had a high survival rate (>92%) on Herculex and non-Bt maize but significantly lower survival on YieldGard VT PRO and PowerCore. In contrast, the HX-R strain did not survive on Agrisure Viptera and Agrisure Viptera 3. The VT-R strain exhibited larval survival greater than 99% on YieldGard VT PRO, which did not differ from its survival on non-Bt maize. In contrast, this strain had significantly lower survival (less than 75%) on Herculex and PowerCore. The VT-R strain also did not survive on Agrisure Viptera or Agrisure Viptera 3. In contrast, this strain had greater than 98% survival on non-Bt maize. The PW-R strain had similar survival rates on Herculex, YieldGard VT PRO and PowerCore, ranging from 78 to 83%. In contrast, no live larvae of the PW-R strain were found on Agrisure Viptera or Agrisure Viptera 3, but on non-Bt maize, this strain had greater than 96% survival. The Vip-R strain had greater than 83% survival on Agrisure Viptera and Agrisure Viptera 3, which was not different than on Herculex or non-Bt maize. In contrast, the Vip-R strain did not survive on YieldGard VT PRO or PowerCore. With the exception of the heterozygous larvae from HX-R × Sus and Vip-R × Sus, which survived on Herculex, all of the heterozygous larvae suffered complete mortality on all of the Bt maize tested. The Sus strain survived only on non-Bt maize, with 82% survival.

### Survival of FAW strains on Bt cotton varieties

A significant interaction occurred between Bt cotton varieties and FAW strains (df = 24, deviance = 602.86, *P *< 0.0001). The larval survival also differed significantly among the Bt cotton varieties (df = 3, deviance = 2255.18, *P *< 0.0001) and the FAW strains (df = 8, deviance = 427.92, *P *< 0.0001).

**Figure 2:**
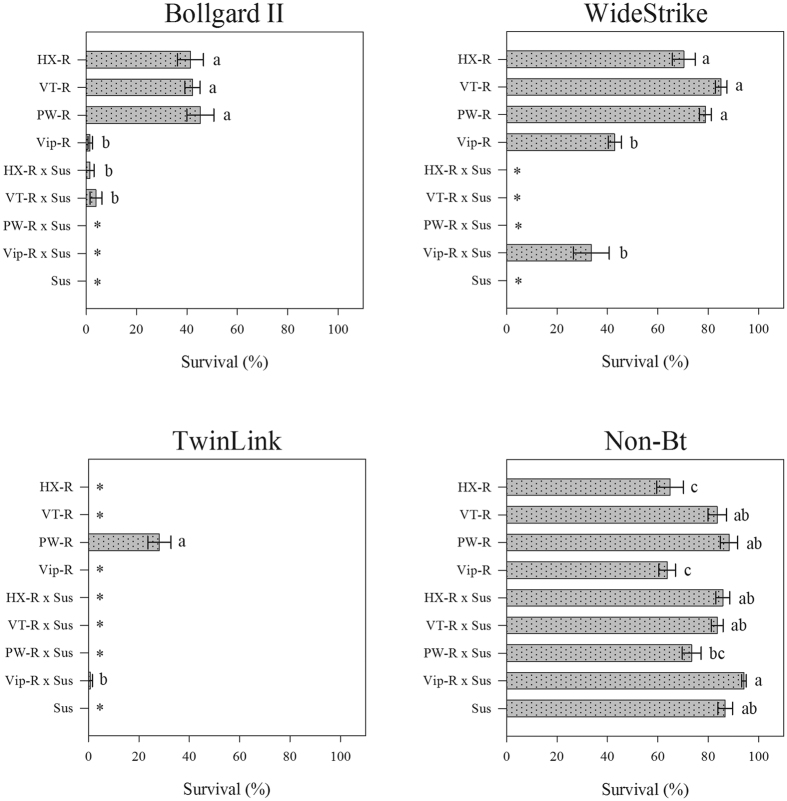
Survival of Bt-resistant, heterozygous and susceptible strains of FAW on leaves of Bt and non-Bt cotton at 7 days. Bars (±SE) with the same letter were not significantly different when the confidence intervals overlap (95% CI). An asterisk (*) indicates that confidence intervals were not estimated because no variability existed.

Figure 2 shows data for the Bt cotton varieties as a fixed effect. On Bollgard II, the HX-R, VT-R, and PW-R strains had similar larval survival, ranging from 41 to 46%. In contrast, less than 4% survival was observed for larvae of the Vip-R, HX-R × Sus and VT-R × Sus strains. On this Bt cotton, the Sus, PW-R × Sus and Vip-R × Sus strains did not survive (Fig. 2). On WideStrike, the HX-R, VT-R and PW-R strains had survival rates greater than 70% and did not differ from each other. In contrast, significantly lower survival was observed for the Vip-R and Vip-R × Sus strains — less than 43%. Larvae from the Sus, HX-R × Sus, VT-R × Sus and PW-R × Sus strains did not survive on WideStrike (Fig. 2). On TwinLink, only larvae of the PW-R (28% survival) and Vip-R × Sus strains (1% survival) survived (Fig. 2). Larval survival was greater than 63% for all strains on non-Bt cotton (Fig. 2).

High survival of the FAW strains was found on all Bt cotton varieties tested (Supplementary Table S2). Larvae from the HX-R strain had a high survival on WideStrike and non-Bt cotton — greater than 65%. This strain also exhibited approximately 40% survival on Bollgard II, but it did not survive on TwinLink. The VT-R strain exhibited greater than 83% survival on WideStrike and non-Bt cotton; however, its survival was less than 42% on Bollgard II, and it did not survive on TwinLink. The PW-R strain had a similar survival on WideStrike (78%) and non-Bt cotton (88%), but a higher survival rate on Bollgard II (45%) and TwinLink (28%). The Vip-R strain larvae showed 43% and 1.6% survival on WideStrike and Bollgard II, respectively; however, a higher survival rate of approximately 64% was detected on non-Bt cotton. Heterozygous Vip-R × Sus and VT-R × Sus larvae survived on WideStrike and Bollgard II, but less than 2% survival was observed for larvae from the Vip-R × Sus strain exposed to Bollgard II. Neonates from the Sus strain did not survive on any Bt cotton variety tested, but their survival was greater than 86% on non-Bt cotton.

## Discussion

The Bt-resistant FAW strains (HX-R, VT-R, PW-R, and Vip-R) had high survival on the studied Bt maize varieties to which they were known to be resistant. In contrast, the Bt-susceptible FAW strain (Sus) showed complete mortality on all Bt maize and cotton varieties tested. These results demonstrate that Bt resistance is largely genetic, as found in previous studies^6^,^20^,^21^,^30^. In addition, the HX-R strain also survived on YieldGard VT PRO and PowerCore maize, and the VT-R and PW-R strains survived on Herculex maize. In contrast, the Vip-R strain only survived on Agrisure Viptera and Agrisure Viptera 3 maize. The survival of the HX-R strain on YieldGard VT PRO and PowerCore maize and of the VT-R and PW-R strains on Herculex maize is due to cross-resistance between the Cry1F and Cry1A.105 proteins. This hypothesis is supported by previous studies that showed a high resistance ratio of Cry1F-resistant strains when exposed to Cry1A.105^12^,^13^,^14^,^16^. This cross-crop resistance occurs due to the similar amino acid sequences of Cry1F and Cry1A.105 proteins, with 72% similarity^26^. Additionally, the Cry1 proteins also share the same binding site in the midgut of the FAW larvae^15^. The YieldGard VT PRO and PowerCore maize varieties also express Cry2Ab2, which has ~35 and 34% similarity with Cry1F and Cry1A.105, respectively, but does not share the same binding site with the Cry1 proteins^15^,^26^. However, expression of Cry2Ab2 did not cause complete mortality of the FAW strains resistant to Cry1F and/or Cry1A.105 due to the low toxicity of this Bt protein to FAW^12^,^16^. We also detected that the Vip-R strain showed complete mortality on YieldGard VT PRO and PowerCore, which indicated no cross-resistance between the Cry1 and Vip3A proteins. This lack of cross-resistance was also previously reported in FAW and tobacco budworm, *Chloridea virescens*^12^,^13^,^14^,^31^,^32^,^33^,^34^. In contrast, the Vip-R strain had a high survival rate on Herculex, but the HX-R strain did not survive on the Agrisure Viptera and Agrisure Viptera 3 maize varieties, indicating the presence of multiple resistances in the Vip-R strain to the Vip3Aa20 and Cry1F proteins. In other words, during the F_2_ screening tests to obtain a Vip3Aa20-resistant strain, the resistance allele to the Cry1F protein was also selected. This probably occurred due to a high frequency of the resistance allele to the Cry1F protein in Brazilian populations of FAW, as reported by Farias *et al.*^35^,^36^.

We also observed that all Bt-resistant FAW strains had a relatively high survival rate on the Bollgard II and WideStrike cotton varieties, whereas the PW-R strain showed some survival on TwinLink cotton. The survival of FAW on Bollgard II and WideStrike is due to the low toxicity and low expression of Cry1Ac against FAW^37^,^38^,^39^,^40^. In addition, the survival of FAW on Bollgard II can also be explained by the low toxicity of Cry2Ab2 against this species^12^,^16^; however, on WideStrike, the resistance of FAW was due to field-evolved resistance to Cry1F^6^,^33^. In contrast, the HX-R, VT-R, and Vip-R strains did not survive on TwinLink, which expresses Cry1Ab and Cry2Ae. In this Bt cotton variety, the Cry1Ab protein did not cause high larval mortality of FAW^9^,^32^,^33^,^41^,^42^. The tolerance of this species to Cry1Ab can be explained by its low affinity for the midgut tissue and more rapid degradation in the larval midgut^43^,^44^. Therefore, the mortality of FAW on TwinLink is basically caused by the expression of the Cry2Ae protein, which has a different binding site from the Cry1 proteins and relatively high toxicity against FAW^45^. In contrast, the survival of the PW-R strain on TwinLink is due to the low toxicity of Cry1Ab and cross-resistance between the Cry2Ab and Cry2Ae proteins, as reported by Gouffon *et al.*^45^.

Our results also showed that all of the heterozygous FAW strains had complete mortality on the YieldGard VT PRO, PowerCore, Agrisure Viptera, and Agrisure Viptera 3 maize varieties. These results demonstrate that the *in-plant* expression of the Bt proteins meets the definition of high-dose, which causes the resistance to be ‘functionally’ recessive. The mortality of the heterozygous strains supports the adoption of refuge areas for resistance management of FAW in Brazil. In contrast, larvae from the reciprocal crosses HX-R × Sus and Vip-R × Sus survived on Herculex maize. The survival of the HX-R × Sus strain on Herculex maize was expected because this Bt maize variety does not meet the definition of high-dose against FAW^6^,^46^. In contrast, the unexpected survival of the Vip-R × Sus strain on Herculex maize is due to the multiple resistances of the Vip-R strain, as discussed above.

The heterozygous HX-R × Sus and VT-R × Sus larvae survived on the Bollgard II cotton, whereas Vip-R × Sus had some survival on WideStrike and TwinLink. The survival of these heterozygous strains on the Bt cotton varieties presents a consistent risk for the evolution of resistance because these insects are mainly responsible for carrying the resistance alleles^23^,^47^. The survival of the FAW heterozygotes on Bt cotton varieties can increase the frequency of resistance alleles to Bt proteins expressed on Bt maize varieties and vice-versa. The survival of the FAW heterozygotes on Herculex maize and the low adoption of refuge areas were the primary contributors to the field-evolved resistance to the Cry1F protein in Brazil^6^. In other words, resistance to the Cry1 maize varieties can compromise the efficacy of WideStrike cotton, as demonstrated in our study and as was also shown by Yang *et al.*^33^.

In the current Brazilian crop production system, Bt maize and cotton varieties are grown simultaneously in adjacent areas. The same FAW populations infest both crops in these areas^17^. Several other aspects also increase the selection pressure for resistance to Bt crops in the FAW population, such as (i) multiple crop seasons per year, (ii) a high rate of adoption of Bt crops, (iii) low compliance with refuge areas, (iv) cross-resistance among the Cry1 proteins, (v) several generations of FAW per year, and (vi) the high reproductive capacity of FAW. In this scenario, FAW developed resistance to Cry1F and Cry1Ab^6^,^18^, and if the same conditions prevailed, resistance to other Bt proteins or Bt crops would also quickly develop. In this study, we demonstrated that the functional dominance of FAW resistance to Bt proteins is recessive due to the high mortality of the heterozygous insects on Bt maize and cotton varieties. Therefore, to support the high-dose refuge strategy, increasing the adoption of refuge areas by farmers is a key point that requires the stakeholders’ urgent attention in an attempt to delay or prevent the evolution of resistance in the FAW population and to prolong the lifetime of the use of Bt crops for controlling this species in Brazil.

## Methods

### Fall armyworm strains

#### Herculex-resistant strain (HX-R)

The strain resistant to Herculex maize, which expresses the Cry1F protein, was selected from a field population collected in Barreiras, Bahia, Brazil, as described in detail by Farias *et al.*^6^. This strain was subsequently used in eight repeated back crossings, each followed by sib-mating and selection among susceptible and resistant insects on diet-overlay bioassays with the Cry1F protein to obtain a nearly isogenic resistant strain^30^. The resistance ratio to the Cry1F protein was greater than 5000-fold, and the inheritance of resistance was characterized as autosomal^6^.

#### YieldGard VT PRO-resistant (VT-R) and PowerCore-resistant (PW-R) strains

The strain resistant to the YieldGard VT PRO (Cry1A.105 and Cry2Ab2) and the PowerCore (Cry1A.105, Cry2Ab2 and Cry1F) maize varieties was selected in the laboratory from positive F_2_ screening tests of field populations as described by Bernardi *et al.*^16^,^20^. After selection, the resistant strains were reared for nine generations on the respective maize event. The resistance ratios of the VT-R and PW-R strains to the Cry1F, Cry1A.105 and Cry2Ab2 proteins were greater than 2700-, 3300- and 10-fold, respectively. The inheritance of the resistance was characterized as autosomal recessive and polygenic^16^,^20^.

#### Agrisure Viptera-resistant strain (Vip-R)

The Vip-R strain resistant to the Agrisure Viptera (Vip3Aa20) and Agrisure Viptera 3 (Cry1Ab/Vip3Aa20) maize varieties was selected via an F_2_ screening of a field population from Correntina, Bahia, Brazil^21^,^48^. After selection, the Vip-R strain was reared for seven generations on Agrisure Viptera maize leaves. The resistance ratio to Vip3Aa20 was greater than 3200-fold, and the inheritance of resistance was characterized as autosomal recessive and monogenic^21^.

#### Susceptible strain

The susceptible strain was obtained from EMBRAPA Milho e Sorgo, Sete Lagoas, Minas Gerais, Brazil, has been maintained in the laboratory since 1995 and was free of selection pressure by insecticides and Bt proteins.

#### Heterozygous strains

The heterozygous strains were obtained from a reciprocal cross of resistant♀ × Sus♂, with at least 40 pairs. In the bioassays, only the F_1_ progeny of these crosses were used because inheritance of resistance is not sex-linked in any of the resistant strains, and the heterozygous insects showed similar mortality and growth inhibition responses to the Sus strain in diet-overlay bioassays^6^,^16^,^20^,^21^.

### Bt plants

Five Bt maize and three Bt cotton varieties that are commercially used in Brazil were tested (Table 1). Non-Bt maize and cotton plants were use as control treatments. Maize and cotton plants were cultivated under field conditions at a density of 10 plants per linear meter with 0.5 m between rows. Before the bioassays, all plants were tested for the expression of insecticidal proteins using detection kits (Envirologix, QuickStix™).

### Leaf-bioassays

Bioassays were performed with plants from the V_4_ to V_8_ (maize) and squaring (cotton) phenological stages. Maize and cotton bioassays were performed by taking leaves from the corn whorls or the upper third of the plants, respectively. In the laboratory, the leaves were cut into ~4-cm^2^ pieces and placed in a gelled mixture of 2% agar-water in 32-well plastic plates (Advento do Brasil, São Paulo, Brazil). Then, each well was infested with one neonate (<24 h old). The plates were sealed with their plastic covers and placed in a room at 27 ± 1 °C and 60 ± 10% RH, with a photoperiod of 14 h. A completely randomized experimental design was used, with 128 larvae/strain/Bt plant (4 replicates of 32 neonates). The leaves were replaced every three days. The larval survival was assessed at 7 days.

### Statistical analysis

A binomial generalized linear model was fit to the larval survival data with a linear interaction predictor (Bt plant *vs* insect genotype). The goodness-of-fit was evaluated using half-normal plots with a simulated envelope^49^. An analysis of deviance was performed to assess the significance of the interaction between the factors (i.e., the Bt plant and insect genotypes) and whether the effect was non-significant (*P *= 0.05). All of these analyses were performed using R^50^, but the means were compared via the confidence intervals (95%) for the linear predictors of the fitted model. The CIs were obtained using the PROC GENMOD (link function = logit) procedure in SAS 9.3^51^. Using the OUTPUT statement, it was possible to obtain the fitted values and their confidence intervals, using the option command output out = pred p = predi, lower = lcl upper = ucl, but the tables and figures contain the original means.

## Additional Information

**How to cite this article**: Horikoshi, R. J. *et al.* Effective dominance of resistance of *Spodoptera frugiperda* to Bt maize and cotton varieties: implications for resistance management. *Sci. Rep.* **6**, 34864; doi: 10.1038/srep34864 (2016).

## Supplementary Material

Supplementary Information

## Figures and Tables

**Table 1:** The Bt maize and cotton varieties tested.

Variety or hybrid	Commercial name	Event	Bt protein
**Maize**			
2B587	Herculex	TC1507	Cry1F
2B587	PowerCore	MON89034 + TC1507	Cry1A.105/Cry2Ab2/Cry1F
DKB 390	YieldGard VT PRO	MON89034	Cry1A.105/Cry2Ab2
Maximus	Agrisure Viptera	MIR162	Vip3Aa20
Maximus	Agrisure Viptera 3	Bt11 + MIR162	Cry1Ab/Vip3Aa20
2B587	Non-Bt maize	—	—
**Cotton**			
FM 975	WideStrike	281-24-236 + 3006-210-23	Cry1Ac/Cry1F
DP 1228	Bollgard II	MON15985	Cry2Ab2/Cry1Ac
FM 940	TwinLink	T304-40 + GHB119	Cry1Ab/Cry2Ae
FM 993	Non-Bt cotton	—	—
