# Supplementary material for: Effective dominance of resistance of Spodoptera frugiperda to Bt maize and cotton varieties: implications for resistance management
Source: Sci Rep. 2016 Oct 10;6:34864. doi: 10.1038/srep34864 (PMC5056508; doi:10.1038/srep34864)
Supplement: Supplementary Information [file srep34864-s1.pdf]

Effective dominance of resistance of *Spodoptera frugiperda* to Bt maize and cotton varieties: implications for resistance management

Renato J. Horikoshi[1], Daniel Bernardi[1], Oderlei Bernardi[1], José B. Malaquias[1], Daniela M. Okuma[1], Leonardo L. Miraldo[1], Fernando S. de A. e Amaral[1] & Celso Omoto[1,*]

[1]Department of Entomology and Acarology, Luiz de Queiroz College of Agriculture (ESALQ), University of São Paulo (USP), Av. Pádua Dias 11, Piracicaba 13418-900, São Paulo, Brazil. Correspondence and requests for materials should be addressed to RJH (e-mail: rjhorikoshi@gmail.com) or CO (e-mail: celso.omoto@usp.br)

Bt maize[1,2]	Bt-resistant strains of FAW				Heterozygous strains of FAW				Susceptible strain of FAW
	HX-R	VT-R	PW-R	Vip-R	HX-R × Sus	VT-R × Sus	PW-R × Sus	Vip-R × Sus	Sus
Herculex	96.9 ± 1.8 a	64.1 ± 2.0 b	80.5 ± 6.3 b	73.4 ± 3.3 a	1.6 ± 0.9 b	0.0 ± 0.0*	0.0 ± 0.0*	7.8 ± 2.7 b	0.0 ± 0.0*
YieldGard VT PRO	10.2 ± 0.8 c	99.2 ± 0.8 a	78.1 ± 6.5 b	0.0 ± 0.0*	0.0 ± 0.0*	0.0 ± 0.0*	0.0 ± 0.0*	0.0 ± 0.0*	0.0 ± 0.0*
PowerCore	7.81 ± 3.3 d	75.0 ± 5.6 b	82.8 ± 4.3 b	0.0 ± 0.0*	0.0 ± 0.0*	0.0 ± 0.0*	0.0 ± 0.0*	0.0 ± 0.0*	0.0 ± 0.0*
Agrisure Viptera	0.0 ± 0.0*	0.0 ± 0.0*	0.0 ± 0.0*	83.6 ± 4.8 a	0.0 ± 0.0*	0.0 ± 0.0*	0.0 ± 0.0*	0.0 ± 0.0*	0.0 ± 0.0*
Agrisure Viptera 3	0.0 ± 0.0*	0.0 ± 0.0*	0.0 ± 0.0*	85.9 ± 2.7 a	0.0 ± 0.0*	0.0 ± 0.0*	0.0 ± 0.0*	0.0 ± 0.0*	0.0 ± 0.0*
Non-Bt maize	92.2 ± 3.0 b	98.4 ± 0.9 a	96.1 ± 1.5 a	82.0 ± 2.9 a	98.4 ± 1.6 a	94.5 ± 2.7 a	87.5 ± 5.6 a	86.7 ± 2.3 a	82.0 ± 6.0 a

Table S1. Larval survival of Bt-resistant, heterozygous, and susceptible strains of FAW on leaves of Bt and non-Bt maize at 7 days.

[1]Means ± SE in each column followed by the same letter were not significantly different due to confidence interval overlap (95% CI).

[2]An asterisk (*) indicates that the confidence intervals were not estimated because no variability existed.

Bt cotton[1,2]	Bt-resistant strains of FAW				Heterozygous strains of FAW				Susceptible strain of FAW
	HX-R	VT-R	PW-R	Vip-R	HX-R × Sus	VT-R × Sus	PW-R × Sus	Vip-R × Sus	Sus
WideStrike	70.3 ± 4.5 a	85.2 ± 2.3 a	78.9 ± 2.3 a	42.9 ± 2.7 b	0.0 ± 0.0*	0.0 ± 0.0*	0.0 ± 0.0*	33.6 ± 7.2 b	0.0 ± 0.0*
Bollgard II	41.4 ± 5.2 b	42.2 ± 3.0 b	45.3 ± 5.3 b	1.6 ± 0.9 c	1.6 ± 1.2 b	3.9 ± 2.3 b	0.0 ± 0.0*	0.0 ± 0.0*	0.0 ± 0.0*
TwinLink	0.0 ± 0.0*	0.0 ± 0.0*	28.1 ± 4.6 c	0.0 ± 0.0*	0.0 ± 0.0*	0.0 ± 0.0*	0.0 ± 0.0*	0.8 ± 0.7 c	0.0 ± 0.0*
Non-Bt cotton	64.8 ± 5.3 a	83.6 ± 3.7 a	88.3 ± 3.5 a	63.7 ± 3.3 a	85.8 ± 2.7 a	83.6 ± 2.3 a	73.4 ± 3.7 a	94.2 ± 0.9 a	86.7 ± 2.9 a

Table S2. Larval survival of Bt-resistant, heterozygous, and susceptible strains of FAW on leaves of Bt and non-Bt cotton after 7 days. [1]Means ± SE in each column followed by the same letter were not significantly different due to confidence interval overlap (95% CI). [2]An asterisk (*) indicates that the confidence intervals were not estimated because no variability existed.
